# Rapid Clinical Response to Omalizumab Treatment in Pediatric Acute Urticaria Associated with Mycoplasma Infection: A Two-Case Report

**DOI:** 10.3390/pediatric18010002

**Published:** 2025-12-19

**Authors:** Zhen-Li Wu, Yi-Siang Huang, Chien-Ting Chiang, Hong-Ren Yu

**Affiliations:** 1 Kaohsiung Chang Gung Memorial Hospital, Kaohsiung 833, Taiwan; dpluszr52@gmail.com; 2 College of Medicine, Chang Gung University, Taoyuan 330, Taiwan; 3 Department of Pediatrics, Kaohsiung Chang Gung Memorial Hospital, Kaohsiung 833, Taiwan; hiss2223@gmail.com; 4 Department of Leisure and Sport Management, Cheng Shin University, Kaohsiung City 833301, Taiwan; 5 Institute for Translational Research in Biomedicine, Chang Gung Memorial Hospital, Kaohsiung 833, Taiwan

**Keywords:** mycoplasma, acute urticaria, omalizumab, children

## Abstract

**Background and Clinical Significance**: Urticaria in children is generally self-limiting, and infections are a significant trigger. While anti-IgE therapy (Omalizumab) is approved for chronic spontaneous urticaria (CSU) in adolescents and adults, its role in treating acute urticaria, particularly in children, is not well defined. **Case Presentation**: We present two pediatric cases of acute urticaria associated with *Mycoplasma pneumoniae* infection. Both cases were refractory to antihistamines and corticosteroids but showed rapid response with anti-IgE treatment. **Conclusions**: This is the first case report in the literature of pediatric acute urticaria treated with Omalizumab. These cases suggest a potential role for IgE-mediated pathways in acute urticaria related to Mycoplasma infection and raise the question of broader applications for Omalizumab beyond CSU.

## 1. Introduction

Urticaria is a common dermatological issue in the pediatric population. Acute urticaria (AU), defined as symptoms lasting less than six weeks, is often triggered by infections, allergens, or medications. Chronic urticaria (CU), lasting more than six weeks, may be idiopathic or autoimmune. Among infectious triggers, *Mycoplasma pneumoniae* is known to contribute to both acute and chronic forms of urticaria [1,2]. Standard treatment for AU includes antihistamines and short-term corticosteroids for more severe cases [1]. Omalizumab, a monoclonal anti-IgE antibody, is currently FDA-approved for chronic spontaneous urticaria (CSU) in patients over 12 years old but is not indicated for acute urticaria. We report two pediatric cases of Mycoplasma-associated AU, both of which showed a favorable response to off-label use of Omalizumab.

## 2. Case Presentation

### 2.1. Case One

**Figure 1:**
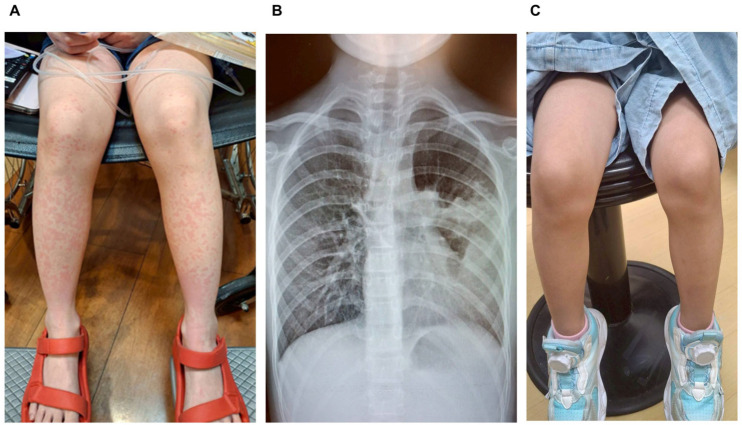
(**A**) Representative photograph of the severe, widespread, pruritic urticaria affecting the face, trunk, and extremities, shown here on the lower extremities, before Omalizumab treatment. (**B**) The Chest X-ray shows a prominent consolidation in the left mid and lower lung lobes, characterized by the presence of an air bronchogram sign. (**C**) After Omalizumab treatment, the urticaria resolved rapidly the following day.

A previously healthy 4-year and 5-month-old girl presented on 26 May 2025, with an 8-day history of fever and cough, followed by a truncal rash that developed one day before admission (Figure 1A). She had been treated twice at local clinics with amoxicillin, but her fever persisted. Upon evaluation in the pediatric emergency department, her vital signs were as follows: temperature 37.3 °C, heart rate 140/min, respiratory rate 25/min, and blood pressure 89/56 mmHg. Physical examination revealed an ill appearance, pharyngeal injection, and an erythematous rash on the trunk. The Glasgow Coma Scale score was E4V5M6. Laboratory investigations indicated a markedly elevated CRP of 80.54 mg/L, and chest radiography showed right upper lobe consolidation (Figure 1B). A presumptive diagnosis of pneumonia and acute urticaria was made. She was admitted and started on intravenous ceftriaxone, azithromycin, and supportive care, including cetirizine, intravenous (IV) diphenhydramine, and IV hydration. By hospital day 3, her CRP had declined to 28.47 mg/L, and *Mycoplasma pneumoniae* IgM tested positive. Despite improvement in fever and cough, a new, extensive urticarial rash developed on the face, hands, and thighs. Suspected to be related to mycoplasma infection, the patient was treated with intravenous hydrocortisone (Table 1). However, three days later, the rash and itching had not improved. After discussing with her family, the child received a single subcutaneous dose of 150 mg of Omalizumab (brand name Xolair) on the eighth hospital day. Following Omalizumab treatment, the rash rapidly resolved the next day (Figure 1C), and the patient was discharged home. A follow-up visit a week later revealed a minor recurrence of urticaria, which resolved with oral antihistamines. The patient was monitored for any adverse drug reactions and remained symptom-free with no signs of severe complications during the subsequent 6-month follow-up period.

### 2.2. Case Two

**Figure 2:**
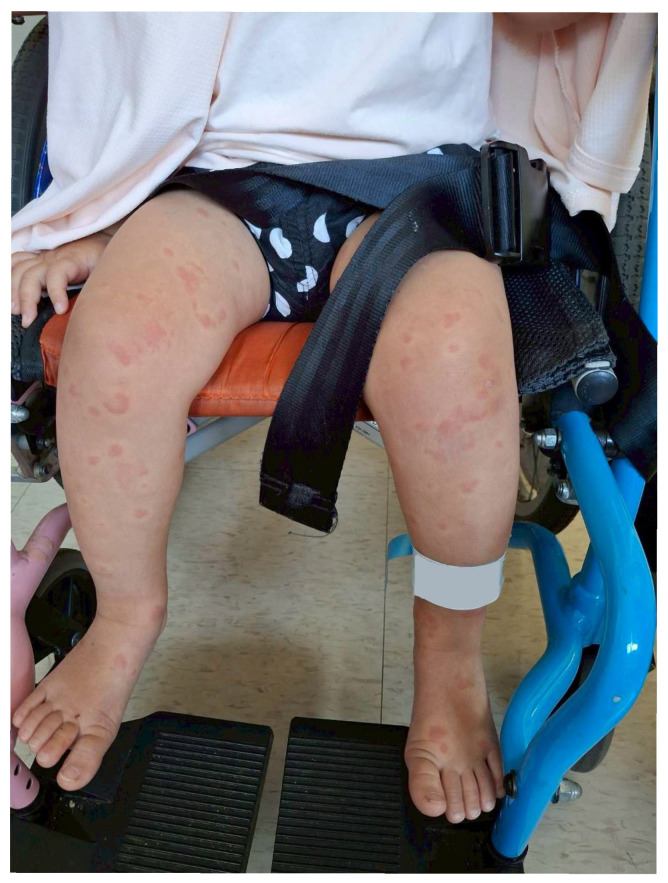
Representative photograph of the severe, widespread, pruritic urticaria affecting the face, trunk, and extremities, shown here on the lower extremities, before Omalizumab treatment.

A 1-year and 8-month-old girl developed diffuse erythematous itching plaques (Figure 2) after her third Enterovirus vaccination, accompanied by cough, rhinorrhea, and vomiting on 13 June 2025. Laboratory findings included leukocytosis (WBC: 12.9 × 10^3^/μL), a CRP level of 12.7 mg/L, and an elevated Mycoplasma IgM titer (1240.6 U/mL) (Table 2). Initial treatment with antihistamines and intravenous hydrocortisone, along with azithromycin (for Mycoplasma infection), proved ineffective. She had no prior history of allergies or adverse reactions to previous vaccinations. Four days after the onset of symptoms, due to worsening urticaria, omalizumab (Xolair) 150 mg was administered off-label. Within 48 h, the rash markedly improved, and she was subsequently discharged in stable condition. Short-term follow-up confirmed complete resolution of the urticaria with no recurrence or signs of adverse events related to Omalizumab administration. During the subsequent three-month follow-up period, only minor recurrences of urticaria were noted, all of which promptly resolved with oral antihistamines. No serious long-term side effects were observed. A recent telephone follow-up in November 2025 confirmed that the patient has had no further episodes of urticaria.

## 3. Discussion

Infectious causes account for 40–60% of urticaria cases in children, with *Mycoplasma pneumoniae* being one of the well-documented pathogens [2,3,4]. Other common triggers include upper respiratory viruses, EBV, drugs, and foods. AU is usually self-limiting and managed with antihistamines [1]. CU may involve autoimmune mechanisms such as IgG autoantibodies against FcεRI or IgE [3]. Omalizumab is approved for the treatment of CSU [1]. It binds to free IgE, preventing it from activating mast cells and basophils, thereby reducing the frequency and severity of CSU flares [2,5].

*Mycoplasma pneumoniae* can provoke urticaria through immune complex deposition, IgE overproduction, or proinflammatory cytokine release (e.g., IL-6, IL-8) [3]. Such cases may mimic autoimmune urticaria in clinical behavior and immune activation profile, but typically resolve with targeted antibiotic therapy. While omalizumab is not approved for AU with identifiable causes (e.g., infections), its efficacy in downregulating IgE pathways provides a theoretical basis for broader use. The rapid resolution of symptoms in our case following omalizumab administration suggests the possibility of IgE-dependent pathways involved in this acute infection-triggered urticaria. Although speculative, elevated baseline IgE or infection-induced IgE sensitization could underlie the treatment response [6].

We acknowledge the possibility of co-existing sensitization in Case 1, as reflected by the elevated total IgE (1357 KU/L). However, due to the acute infectious process, a comprehensive allergy workup was not prioritized. We did not perform parasitology assessment as there were no clinical or epidemiological indications (e.g., peripheral eosinophilia, specific travel history) suggestive of parasitic infection. While high total IgE can be an incidental finding or a non-specific response to infection, the prompt and dramatic resolution of symptoms following anti-IgE therapy strongly suggests that the IgE/mast cell pathway was a critical and targetable effector mechanism driving the severity of the refractory urticaria in these cases, irrespective of the initial trigger (infection or potential sensitization). Regarding Case 2, where Total IgE was within the normal range (32.6 KU/L), the rapid response to omalizumab supports the notion that IgE concentration is not the sole determinant of treatment efficacy in refractory disease. Formal drug challenge tests were not performed due to the acute instability of the patient.

We recognize the validity of validated tools for chronic urticaria, such as the 7-day urticaria activity score (UAS7) and the urticaria control test (UCT). However, due to the acute nature of the condition, the rapid progression of the disease, and the inability of young children to adequately express their itching, these tools, primarily used for tracking chronic urticaria, are not suitable for treatment decisions in these two cases. The designation of ‘refractory’ was clinically determined by the failure of widespread, pruritic urticaria to respond after several days of maximum-dose antihistamines and high-dose intravenous corticosteroids. We recognize the critical nature of using omalizumab in such young patients as an off-label treatment. This decision was made only after a thorough evaluation of the risks and benefits, given the severity of the refractory symptoms and lack of response to standard therapy. The treatment was carried out under Institutional Review Board (IRB) approval and with the explicit written informed consent of the patient’s guardians for both the administration of the drug and the publication of this report.

We confirm that the omalizumab administered in both cases was the original reference product, Xolair, to ensure treatment consistency and adherence to known efficacy profiles. The safety profile of anti-IgE therapy in very young children remains a key consideration. In both presented cases, the single subcutaneous dose of omalizumab (150 mg) was well-tolerated, with no immediate hypersensitivity reactions, injection site reactions, or other acute severe adverse events observed during hospitalization and immediate follow-up. This rapid and safe clinical response is encouraging, though long-term data on children under 12 remains limited.

## 4. Conclusions

These two pediatric cases underscore the clinical relevance of *Mycoplasma pneumoniae* as a trigger for acute urticaria. This is the first case report in the literature of pediatric acute urticaria treated with Omalizumab. The dramatic response to Omalizumab in these two pediatric cases challenges current treatment paradigms and highlights a potential overlap in pathophysiological mechanisms between acute infectious urticaria and CSU. Given the off-label use in this young population, we strongly emphasize that medium- and long-term follow-up (e.g., up to one year) is essential to fully assess the sustained efficacy and long-term safety of anti-IgE therapy and to monitor for the potential occurrence of future drug resistance. Prospective studies are warranted to assess the safety and efficacy of anti-IgE therapy in selected cases of refractory acute urticaria, particularly those with infectious etiologies.

## Figures and Tables

**Table 1:** The baseline characteristics, laboratory data, and clinical course of the first case.

Case	Sex/Age, Wt (yr/Kg)	Presentations	Total-IgE (0–100 KU/L)	White Blood Cell Count (4–10 × 10^9^/L)	Neutrophil Count (2–7 × 10^9^/L)	C-Reactive Protein (0–8 mg/L)	Treatment Before Omalizumab
1	F/4Y5M, 17.8	Wheals, swelling over trunk	1357	9.01	6.06	80.54	Hydrocortisone 300 mg every day for 5 days

Wt: body weight; F: female.

**Table 2:** The baseline characteristics, laboratory data, and clinical course of the second case.

Case	Sex/Age, Wt (yr/Kg)	Presentations	Total-IgE (0–100 KU/L)	White Blood Cell Count (4–10 × 10^9^/L)	Neutrophil Count (2–7 × 10^9^/L)	C-reactive Protein (0–8 mg/L)	Treatment Before Omalizumab
2	F/1Y8M, 10	Wheals, swelling over trunk and extremities	32.6	12.9	7.998	12.7	Hydrocortisone 200 mg per day for 4 days

Wt: body weight; F: female.

## Data Availability

The data that supports the findings of the manuscript may be available from the corresponding author upon reasonable request that takes into account patient privacy requirements.

## Abbreviations

The following abbreviations are used in this manuscript:

AU	Acute urticaria
CU	Chronic urticaria
CSU	Chronic spontaneous urticaria
